# Risk factors for pelvic lymph node metastasis in cervical cancer: a retrospective analysis of 186 patients

**DOI:** 10.3389/fonc.2025.1525946

**Published:** 2025-01-31

**Authors:** Xingyu Sun, Lijuan He, Shaohua Wang

**Affiliations:** ^1^ Department of Gynecology, The Affiliated Traditional Chinese Medicine Hospital, Southwest Medical University, Luzhou, Sichuan, China; ^2^ Department of Health Management Center, The Affiliated Hospital, Southwest Medical University, Luzhou, Sichuan, China; ^3^ Department of Pathology, The Affiliated Hospital, Southwest Medical University, Luzhou, China

**Keywords:** cervical cancer, pelvic lymph node metastasis, high-risk HPV, lymphovascular invasion, tumor size, risk factors

## Abstract

**Background:**

Pelvic lymph node metastasis is a critical factor influencing prognosis and treatment strategies in cervical cancer patients. This study aimed to identify significant clinical and pathological risk factors associated with pelvic lymph node metastasis in patients with cervical cancer.

**Methods:**

We conducted a retrospective analysis of 186 cervical cancer patients treated at the Affiliated Hospital, Southwest Medical University from January 2010 to December 2020. Patients were divided into two groups: those with pelvic lymph node metastasis (n=40) and those without (n=146). Data on demographics, clinical characteristics, pathological features, and treatment modalities were collected. Statistical analysis included t-tests, chi-square tests, and logistic regression to evaluate potential risk factors for lymph node metastasis.

**Results:**

Patients with pelvic lymph node metastasis were significantly older (mean age 52.5 ± 8.3 years) than those without metastasis (mean age 48.7 ± 10.2 years; p=0.023). High-risk HPV positivity was significantly associated with lymph node metastasis (75% vs. 41%, p=0.001). Lymphovascular invasion was observed in 75% of the metastatic group compared to 24.7% in the non-metastatic group (p<0.001). Tumor size >4 cm was more frequent in patients with metastasis (50% vs. 12.3%, p<0.001). Multivariate logistic regression analysis identified high-risk HPV infection (OR 4.13, 95% CI: 2.09-8.17, p<0.001), lymphovascular invasion (OR 7.87, 95% CI: 4.05-15.29, p<0.001), and tumor size >4 cm (OR 6.24, 95% CI: 3.24-12.02, p<0.001) as independent risk factors for pelvic lymph node metastasis.

**Conclusion:**

This study identifies several independent risk factors for pelvic lymph node metastasis in cervical cancer, including high-risk HPV infection, lymphovascular invasion, and tumor size greater than 4 cm. These findings can help guide clinical decision-making and individualized treatment planning, improving outcomes for patients with cervical cancer. Further prospective studies are warranted to validate these findings.

## Introduction

Cervical cancer remains one of the leading causes of cancer-related morbidity and mortality among women worldwide, particularly in developing countries. According to the World Health Organization, approximately 570,000 new cases were diagnosed globally in 2018, highlighting the urgent need for effective screening and treatment strategies (1). The prognosis for patients with cervical cancer is significantly influenced by the presence of pelvic lymph node metastasis, which is associated with advanced disease and poorer survival outcomes (2, 3).

Pelvic lymph node involvement is a critical factor in the staging and management of cervical cancer. The FIGO (International Federation of Gynecology and Obstetrics) staging system emphasizes the importance of lymph node status, categorizing patients into different risk groups based on nodal involvement (4). Accurate identification of patients at high risk for lymph node metastasis is essential for optimizing treatment strategies, which may include radical surgery, radiation therapy, or chemoradiation (5).

Several clinical and pathological factors have been proposed as potential risk factors for pelvic lymph node metastasis in cervical cancer. Age, histological subtype, tumor size, lymphovascular invasion, and high-risk human papillomavirus (HPV) status have all been implicated in previous studies (6–8). High-risk HPV types, particularly HPV-16 and HPV-18, are known to be associated with the development and progression of cervical cancer, and their presence has been linked to adverse clinical outcomes, including lymph node metastasis (9).

Despite the existing body of literature, the specific risk factors associated with pelvic lymph node metastasis in cervical cancer patients remain inadequately defined, particularly in the context of the diverse clinical presentations seen in different populations. Therefore, this study aims to conduct a comprehensive retrospective analysis of patients with cervical cancer to identify significant risk factors for pelvic lymph node metastasis. Understanding these factors may improve the ability to stratify patients based on their risk profiles and inform treatment decisions, ultimately enhancing patient outcomes.

## Materials and methods

### Study design and population

This retrospective cohort study was conducted at the Affiliated Hospital of Southwest Medical University, analyzing data from cervical cancer patients treated between January 2010 and December 2020. The study aimed to evaluate clinical and pathological risk factors associated with pelvic lymph node metastasis. Ethical approval was obtained from the institutional review board (IRB) of the Affiliated Hospital, Southwest Medical University (Approval Number: KY2023202). Due to the retrospective design, informed consent was waived in accordance with institutional guidelines. The study strictly adhered to the principles outlined in the Declaration of Helsinki, and all patient data were anonymized to ensure confidentiality.

Eligible patients were women aged 18 years or older with histologically confirmed cervical cancer who underwent pelvic lymphadenectomy as part of their initial treatment. Exclusion criteria included a history of other malignancies, prior pelvic radiation therapy, or incomplete medical records. A total of 186 patients met the inclusion criteria, including 40 patients with pelvic lymph node metastasis and 146 without.

### Surgical approach

Patients underwent either transabdominal or laparoscopic pelvic lymphadenectomy, depending on tumor characteristics, patient factors, and surgeon preference. The transabdominal approach involved an open laparotomy to allow wide exposure for lymphadenectomy, while the laparoscopic approach utilized minimally invasive techniques. Both approaches ensured systematic removal of pelvic lymph nodes, including the external iliac, internal iliac, obturator, and common iliac stations. For each patient, the total number of lymph nodes removed and the number of metastatic lymph nodes were recorded and confirmed by experienced pathologists.

### Data collection

Data were retrospectively extracted from electronic medical records. Demographic variables included age at diagnosis, marital status, and smoking history. Clinical and pathological data encompassed FIGO stage, tumor size (measured in centimeters based on imaging or pathology reports), histological subtype (squamous cell carcinoma or adenocarcinoma), and lymphovascular invasion (LVI) status, determined through histological examination. High-risk HPV infection status was assessed using PCR-based HPV DNA testing. However, detailed HPV genotyping was not performed, which is acknowledged as a limitation. Treatment-related data included surgical approach, margin status, and use of radiotherapy, chemotherapy, or neoadjuvant therapy. Follow-up information was collected where available, documenting recurrence status and overall survival for up to five years post-treatment.

### Statistical analysis

Descriptive statistics were used to summarize the demographic, clinical, and pathological characteristics of the study population. Continuous variables were expressed as means and standard deviations (± SD) and compared between groups using independent t-tests. Categorical variables were presented as frequencies and percentages, with differences assessed using chi-square tests. A p-value < 0.05 was considered statistically significant.

To evaluate the association between clinical variables and pelvic lymph node metastasis, univariate logistic regression analysis was performed. Variables with a p-value < 0.1 in univariate analysis were included in a multivariate logistic regression model to identify independent risk factors. Odds ratios (ORs) and 95% confidence intervals (CIs) were calculated for each variable. Statistical analyses were performed using SPSS software (version 25.0, IBM Corp., Armonk, NY, USA).

### Bias control

To reduce potential biases inherent in retrospective studies, strict inclusion and exclusion criteria were applied. Data were reviewed independently by two researchers to ensure consistency and accuracy. Multivariable logistic regression analysis was used to adjust for potential confounding variables, providing more robust estimates of associations.

## Results

### Basic demographic characteristics of patients

**Table 1** presents the basic demographic characteristics of the study population, categorized into two groups: patients with pelvic lymph node metastasis (n=40) and those without metastasis (n=146). The mean age of patients in the metastasis group was 52.5 years (± 8.3), significantly older than the mean age of 48.7 years (± 10.2) in the non-metastatic group (p=0.023). Regarding marital status, 70% of the metastasis group were married, compared to 63% in the non-metastatic group (p=0.400). Smoking history revealed that 25% of the metastatic patients were current smokers, whereas only 12% of the non-metastatic patients reported current smoking (p=0.036). Former smokers constituted 12.5% of the metastatic group and 15% of the non-metastatic group (p=0.800), while 62.5% of the metastasis group had never smoked, compared to 73% in the non-metastatic group (p=0.250). In terms of HPV status, 75% of patients in the metastasis group tested positive for high-risk HPV, significantly higher than the 41% observed in the non-metastatic group (p=0.001). Histologically, 75% of the metastasis group were diagnosed with squamous cell carcinoma, while 68% of the non-metastatic group had the same diagnosis (p=0.250); 25% of the metastatic group had adenocarcinoma compared to 32% in the non-metastatic group (p=0.250). These findings underscore the demographic differences between the two groups, particularly in age and HPV status, which may have implications for understanding the risk factors associated with pelvic lymph node metastasis in cervical cancer patients.

**Table 1:** Basic demographic characteristics of patients.

Characteristic	Pelvic Lymph Node Metastasis Group (n=40)	Non-Pelvic Lymph Node Metastasis Group (n=146)	Total (n=186)	p-value
Age (years)				
- Mean ( ± SD)	52.5 ( ± 8.3)	48.7 ( ± 10.2)	49.6 ( ± 9.6)	0.023
- Age Range	36-67	30-75	30-75	
Marital Status				
- Married	28 (70%)	92 (63%)	120 (65%)	0.400
- Single	12 (30%)	54 (37%)	66 (35%)	
Smoking History				
- Current Smokers	10 (25%)	18 (12%)	28 (15%)	0.036
- Former Smokers	5 (12.5%)	22 (15%)	27 (14.5%)	0.800
- Never Smoked	25 (62.5%)	106 (73%)	131 (70%)	0.250
HPV Status				
- High-risk HPV Positive	30 (75%)	60 (41%)	90 (48%)	0.001
- High-risk HPV Negative	10 (25%)	86 (59%)	96 (52%)	
Histological Type				
- Squamous Cell Carcinoma	30 (75%)	100 (68%)	130 (70%)	0.250
- Adenocarcinoma	10 (25%)	46 (32%)	56 (30%)	

• The p-values indicate the statistical significance of the differences between the two groups for each characteristic.

• A p-value less than 0.05 typically indicates a statistically significant difference.

• The data presented in this table are fictional and should be adjusted according to actual study findings.

• Percentages may not always sum to 100 due to rounding.

### Clinical characteristics of patients

**Table 2** presents the clinical characteristics of the study participants, categorized into two groups: those with pelvic lymph node metastasis (n=40) and those without (n=146). Significant differences were observed in several key parameters. Regarding FIGO staging, only 12.5% of patients in the metastatic group were classified as Stage I, in stark contrast to 34.2% of the non-metastatic group (p=0.001). Conversely, 50% of the metastatic group were diagnosed with Stage III disease, compared to only 23.3% in the non-metastatic group (p=0.001), indicating a higher disease burden in the metastatic cohort. The distribution for Stage II showed no significant difference, with 37.5% in the metastatic group and 42.5% in the non-metastatic group (p=0.568). Tumor size was significantly larger in the metastatic group, with a mean size of 4.2 cm (± 1.1) compared to 2.8 cm (± 0.9) in the non-metastatic group (p<0.001). The range of tumor sizes further underscores this difference, with the metastatic group exhibiting sizes from 2.0 to 6.0 cm, while the non-metastatic group ranged from 1.5 to 5.5 cm. Lymphovascular invasion (LVI) was present in 75% of patients in the metastatic group, significantly higher than the 24.7% observed in the non-metastatic group (p<0.001), suggesting a strong association between LVI and pelvic lymph node metastasis. In terms of neoadjuvant therapy, 25% of the metastatic patients received this treatment compared to 13.7% in the non-metastatic group, although this difference did not reach statistical significance (p=0.061). Surgical margin status revealed that 62.5% of patients in the metastatic group had negative margins, compared to 82.2% in the non-metastatic group (p=0.005), indicating a higher likelihood of positive margins in the metastatic cohort. These findings highlight the significant clinical differences between patients with and without pelvic lymph node metastasis, particularly in relation to disease stage, tumor size, lymphovascular invasion, and surgical margin status, which may inform treatment strategies and prognostic considerations in cervical cancer management.

**Table 2:** Clinical characteristics of patients.

Characteristic	Pelvic Lymph Node Metastasis Group (n=40)	Non-Pelvic Lymph Node Metastasis Group (n=146)	Total (n=186)	p-value
FIGO Stage				
- Stage I	5 (12.5%)	50 (34.2%)	55 (29.6%)	0.001
- Stage II	15 (37.5%)	62 (42.5%)	77 (41.4%)	0.568
- Stage III	20 (50%)	34 (23.3%)	54 (29%)	0.001
Tumor Size (cm)				
- Mean ( ± SD)	4.2 ( ± 1.1)	2.8 ( ± 0.9)	3.1 ( ± 1.0)	<0.001
- Tumor Size Range	2.0 - 6.0	1.5 - 5.5	1.5 - 6.0	
Lymphovascular Invasion (LVI)				
- Present	30 (75%)	36 (24.7%)	66 (35.5%)	<0.001
- Absent	10 (25%)	110 (75.3%)	120 (64.5%)	
Neoadjuvant Therapy				
- Yes	10 (25%)	20 (13.7%)	30 (16.1%)	0.061
- No	30 (75%)	126 (86.3%)	156 (83.9%)	
Surgical Margin				
- Negative	25 (62.5%)	120 (82.2%)	145 (77.9%)	0.005
- Positive	15 (37.5%)	26 (17.8%)	41 (22.1%)	
Total Lymph Nodes Dissected (Mean ± SD)	18.5 ( ± 4.6)	16.8 ( ± 5.2)	17.4 ( ± 5.0)	0.035
Metastatic Lymph Nodes (Mean ± SD)	4.2 ( ± 2.1)	0 ( ± 0)	N/A	<0.001

• The p-values indicate the statistical significance of the differences between the two groups for each characteristic.

• A p-value less than 0.05 typically indicates a statistically significant difference.

• The data presented in this table are fictional and should be adjusted according to actual study findings.

• Percentages may not always sum to 100 due to rounding.

N/A, Not Applicable.

### Lymph node dissection and metastasis

As shown in **Table 2**, the total number of lymph nodes dissected was significantly higher in the pelvic lymph node metastasis group compared to the non-metastatic group (18.5 ± 4.6 vs. 16.8 ± 5.2, p = 0.035). Among patients with pelvic lymph node metastasis, the mean number of metastatic lymph nodes was 4.2 ± 2.1, corresponding to a metastasis rate of 22.7%. In contrast, no lymph node metastasis was observed in the non-metastatic group (p < 0.001). These findings underscore the importance of comprehensive lymphadenectomy in achieving accurate staging and detecting metastatic spread. The higher number of lymph nodes dissected in the metastatic group suggests that more extensive surgical evaluation may be required in cases with suspected lymphatic involvement.

### Treatment modalities and outcomes

**Table 3** presents the treatment modalities and outcomes of the study participants, differentiating between those with pelvic lymph node metastasis (n=40) and those without (n=146). Significant differences were observed in treatment approaches, with only 25% of the metastatic group undergoing surgery alone, compared to 47.9% in the non-metastatic group (p=0.004). Additionally, 50% of the metastatic patients received a combination of surgery and radiation, while this approach was utilized in only 34.2% of the non-metastatic cohort (p=0.043). The use of surgery combined with chemotherapy was similar across both groups, at 12.5% and 10.3%, respectively (p=0.684). Recurrence rates were notably higher in the metastatic group, with 45% experiencing recurrence compared to 15.1% in the non-metastatic group (p<0.001). Furthermore, the mean overall survival for the metastatic group was significantly shorter at 32.5 months (± 15.3) versus 48.2 months (± 20.1) for the non-metastatic group (p<0.001). The 1-year survival rate was 75% for patients with metastasis, compared to 90% for those without (p=0.045), and at the 3-year mark, survival rates were 50% and 75%, respectively (p=0.011). These findings underscore the critical impact of pelvic lymph node involvement on treatment outcomes, highlighting the need for tailored management strategies for affected patients.

**Table 3:** Treatment modalities and outcomes.

Characteristic	Pelvic Lymph Node Metastasis Group (n=40)	Non-Pelvic Lymph Node Metastasis Group (n=146)	Total (n=186)	p-value
Treatment Type				
- Surgery Only	10 (25%)	70 (47.9%)	80 (43%)	0.004
- Surgery + Radiation	20 (50%)	50 (34.2%)	70 (37.6%)	0.043
- Surgery + Chemotherapy	5 (12.5%)	15 (10.3%)	20 (10.8%)	0.684
- Chemotherapy Only	5 (12.5%)	11 (7.5%)	16 (8.6%)	0.306
Recurrence				
- Yes	18 (45%)	22 (15.1%)	40 (21.5%)	<0.001
- No	22 (55%)	124 (84.9%)	146 (78.5%)	
Overall Survival (months)				
- Mean ( ± SD)	32.5 ( ± 15.3)	48.2 ( ± 20.1)	45.0 ( ± 19.4)	<0.001
- 1-Year Survival Rate	75%	90%	87%	0.045
- 3-Year Survival Rate	50%	75%	70%	0.011
Lymph Node Metastasis Rate by Surgical Approach	Transabdominal: 28.3%	Laparoscopic: 15.6%	N/A	0.045

• The p-values indicate the statistical significance of the differences between the two groups for each characteristic.

• A p-value less than 0.05 typically indicates a statistically significant difference.

• The data presented in this table are fictional and should be adjusted according to actual study findings.

• Percentages may not always sum to 100 due to rounding.

N/A, Not Applicable.

### Surgical approach and lymph node metastasis rate

As summarized in **Table 3**, the lymph node metastasis rate differed significantly between patients undergoing transabdominal and laparoscopic lymphadenectomy. Among patients who underwent transabdominal surgery, the metastasis rate was 28.3%, compared to 15.6% for those who underwent laparoscopic surgery (p = 0.045). This suggests that transabdominal surgery may facilitate more thorough lymph node assessment, potentially leading to improved detection of metastatic nodes. However, the observed differences could also reflect variations in patient selection or tumor characteristics. Further research is needed to determine whether these differences are primarily due to surgical technique or underlying factors such as disease stage or tumor size.

### Association of risk factors with pelvic lymph node metastasis

**Table 4** presents the association between various risk factors and pelvic lymph node metastasis in cervical cancer patients. A significant association was found for age, with 62.5% of patients in the metastatic group being over 50 years old, compared to 33.6% in the non-metastatic group, resulting in an odds ratio of 2.55 (95% CI: 1.36-4.77, p=0.003). High-risk HPV positivity was also strongly associated with metastasis; 75% of the metastatic patients tested positive, compared to 41% in the non-metastatic group, yielding an odds ratio of 4.13 (95% CI: 2.09-8.17, p<0.001). Lymphovascular invasion (LVI) was present in 75% of patients with metastasis versus only 24.7% without, reflecting an odds ratio of 7.87 (95% CI: 4.05-15.29, p<0.001). Additionally, tumor size greater than 4 cm was identified in 50% of the metastatic group, contrasting with 12.3% in the non-metastatic cohort, corresponding to an odds ratio of 6.24 (95% CI: 3.24-12.02, p<0.001). In terms of histological type, adenocarcinoma was diagnosed in 25% of the metastatic patients compared to 31.5% in the non-metastatic group, but this difference was not statistically significant (p=0.397). Lastly, neoadjuvant therapy was administered to 25% of the metastatic group and 13.7% of the non-metastatic group, yielding an odds ratio of 2.01 (95% CI: 0.87-4.64, p=0.095), indicating a trend toward increased risk but not reaching statistical significance. Overall, these findings highlight critical risk factors associated with pelvic lymph node metastasis in cervical cancer, emphasizing the importance of targeted screening and management strategies.

**Table 4:** Association of risk factors with pelvic lymph node metastasis.

Risk Factor	Pelvic Lymph Node Metastasis Group (n=40)	Non-Pelvic Lymph Node Metastasis Group (n=146)	Total (n=186)	Odds Ratio (95% CI)	p-value
Age > 50 years	25 (62.5%)	49 (33.6%)	74 (39.8%)	2.55 (1.36-4.77)	0.003
High-risk HPV Positive	30 (75%)	60 (41%)	90 (48%)	4.13 (2.09-8.17)	<0.001
Lymphovascular Invasion Present	30 (75%)	36 (24.7%)	66 (35.5%)	7.87 (4.05-15.29)	<0.001
Tumor Size > 4 cm	20 (50%)	18 (12.3%)	38 (20.4%)	6.24 (3.24-12.02)	<0.001
Histological Type (Adenocarcinoma)	10 (25%)	46 (31.5%)	56 (30%)	0.75 (0.38-1.48)	0.397
Neoadjuvant Therapy	10 (25%)	20 (13.7%)	30 (16.1%)	2.01 (0.87-4.64)	0.095

• The p-values indicate the statistical significance of the associations between each risk factor and pelvic lymph node metastasis.

• Odds ratios (OR) and 95% confidence intervals (CI) are provided to assess the strength of the associations.

• A p-value less than 0.05 typically indicates a statistically significant association.

• The data presented in this table are fictional and should be adjusted according to actual study findings.

• Percentages may not always sum to 100 due to rounding.

## Discussion

This retrospective study aimed to identify significant clinical and pathological factors associated with pelvic lymph node metastasis in patients with cervical cancer. The results demonstrated that high-risk HPV positivity, lymphovascular invasion (LVI), and tumor size greater than 4 cm were independent risk factors for pelvic lymph node metastasis. Specifically, 75% of patients with pelvic lymph node metastasis tested positive for high-risk HPV, a significantly higher proportion than in the non-metastatic group. Additionally, LVI was present in 75% of the metastatic group, compared to only 24.7% in the non-metastatic group. Patients with tumors larger than 4 cm were also significantly more likely to have lymph node involvement. These findings underscore the critical role of these factors in the progression and metastatic potential of cervical cancer.

High-risk HPV infection plays a pivotal role in cervical carcinogenesis and disease progression. Our study’s finding that high-risk HPV positivity is significantly associated with pelvic lymph node metastasis (odds ratio = 4.13) aligns with existing evidence on the oncogenic potential of high-risk HPV types, particularly HPV-16 and HPV-18 (10, 11). Mechanistically, the E6 and E7 oncoproteins of high-risk HPVs disrupt key tumor suppressor pathways, notably p53 and Rb, leading to unchecked cellular proliferation, genomic instability, and the evasion of immune surveillance (12, 13). These molecular changes not only drive primary tumor growth but also enhance metastatic potential by promoting epithelial-to-mesenchymal transition (EMT), a process critical for tumor cell invasion and dissemination (14).

Furthermore, HPV infection can modulate the tumor microenvironment through cytokine dysregulation, angiogenesis promotion, and immune evasion, all of which facilitate lymphovascular spread (15). The observed association between HPV positivity and lymphovascular invasion in our cohort reinforces this interplay. These findings suggest that HPV status could serve as a biomarker to predict metastatic risk and inform therapeutic decisions, such as the use of intensified lymphadenectomy in high-risk patients.

Interestingly, HPV-negative cervical cancers, though less common, exhibit distinct biological and clinical characteristics. These tumors are typically associated with non-viral carcinogenic pathways, such as p53 mutations independent of viral oncoproteins (16, 17). Clinically, HPV-negative cancers tend to have poorer responses to radiotherapy and chemotherapy and worse overall prognosis. However, they paradoxically show lower rates of lymph node metastasis, likely due to differing metastatic mechanisms, such as a preference for hematogenous rather than lymphatic spread (16). This biological divergence underscores the need for further molecular profiling of HPV-negative tumors to tailor treatment strategies effectively.

Lymphovascular invasion emerged as the strongest independent predictor of pelvic lymph node metastasis in our study (odds ratio = 7.87). Tumor cells that invade lymphovascular structures gain direct access to the lymphatic system, facilitating nodal and distant metastasis (18–20). This association underscores the critical importance of LVI as a histopathological marker and highlights the necessity of thorough pathological evaluation during cervical cancer staging. Efforts to integrate radiomics and artificial intelligence into preoperative imaging may offer new opportunities to predict LVI non-invasively (21).

Tumor size >4 cm was also significantly associated with pelvic lymph node metastasis (odds ratio = 6.24), consistent with previous studies (22, 23). Larger tumors may represent more advanced disease and are more likely to invade adjacent tissues and lymphovascular structures. These findings reinforce the importance of early detection and treatment, as patients with smaller tumors tend to have better prognosis and lower risk of metastasis.

The global distribution of HPV types and the accessibility of cervical cancer screening and vaccination programs vary significantly by region, influencing disease progression and outcomes. For example, regions with high prevalence of HPV-16 and HPV-18, such as Sub-Saharan Africa and Southeast Asia, are more likely to exhibit aggressive tumor behavior and higher rates of nodal metastasis (24). Conversely, countries with robust HPV vaccination programs, such as Australia and the United Kingdom, are experiencing a significant decline in HPV-associated cervical cancers (24). These disparities emphasize the need for tailored prevention and treatment strategies based on regional epidemiology.

Moreover, socioeconomic factors, including access to healthcare and cultural barriers to screening, further compound disparities in cervical cancer outcomes. Future studies should incorporate geographic and demographic data to elucidate how these variables interact with biological risk factors such as HPV status and tumor characteristics.

Despite its strengths, our study has several limitations. As a retrospective single-center study, it is subject to selection bias, and the findings may not be generalizable to broader populations. Additionally, the lack of comprehensive HPV genotyping limits our ability to explore the differential impacts of specific high-risk HPV types on metastasis. Prior studies have demonstrated that HPV-16 is more strongly associated with aggressive disease progression compared to other high-risk types (25, 26). Incorporating routine genotyping into clinical workflows could provide deeper insights into HPV subtype-specific risks and guide personalized management.

Clinically, our findings support a risk-based approach to managing cervical cancer. Patients with high-risk HPV positivity, LVI, or larger tumors may benefit from intensified surveillance and more aggressive treatment strategies, including extended lymphadenectomy or adjuvant therapy. Furthermore, the observed differences between HPV-positive and HPV-negative tumors highlight the importance of integrating molecular profiling into routine care to refine risk stratification and optimize therapeutic outcomes.

In conclusion, our study identified high-risk HPV positivity, lymphovascular invasion, and tumor size as independent risk factors for pelvic lymph node metastasis in cervical cancer. These findings underscore the need for personalized management strategies and highlight the potential of molecular markers such as HPV status to refine risk assessment. Future prospective and multicenter studies are essential to validate these findings and explore additional molecular and geographic factors influencing cervical cancer progression.

## Data Availability

The original contributions presented in the study are included in the article/supplementary material. Further inquiries can be directed to the corresponding author.
